# *Staphylococcus epidermidis* in feedings and feces of preterm neonates

**DOI:** 10.1371/journal.pone.0227823

**Published:** 2020-02-03

**Authors:** Laura Moles, Marta Gómez, Elena Moroder, Gerardo Bustos, Ana Melgar, Rosa del Campo, Juan M. Rodríguez

**Affiliations:** 1 Department of Nutrition and Food Science, Complutense University of Madrid, Madrid, Spain; 2 Pediatrics Sevirce, Hospital Francesc de Borja, Gandía, Valencia, Spain; 3 Neonatology Service, University Hospital 12 de Octubre, Madrid, Spain; 4 Red de Salud Materno-Infantil y del Desarrollo (SAMID), Carlos III Institute, Madrid, Spain; 5 Department of Public Health, Mother and Child, Complutense University of Madrid, Madrid, Spain; 6 Microbiology and Parasitology Service, University Hospital Ramón y Cajal, Ramón y Cajal Health Research Institute, Madrid, Spain; University of Minnesota Twin Cities, UNITED STATES

## Abstract

*Staphylococcus epidermidis* has emerged as the leading agent causing neonatal late-onset sepsis in preterm neonates; although the severity of the episodes caused by this species is often underestimated, it might exert relevant short- and long-term detrimental effects on neonatal outcomes. In this context, the objective of this study was to characterize a collection of *S*. *epidermidis* strains obtained from meconium and feces of preterm infants, and to assess the potential role of the enteral feeding tubes as potential reservoirs for this microorganism. A total of 26 preterm infants were enrolled in the study. Meconium and fecal samples were collected weekly during their first month of life (n = 92). Feeding samples were collected after their pass through the enteral feeding tubes (n = 84). *S*. *epidermidis* was present in the fecal samples of all the infants in, at least, one sampling time at concentrations ranging from 6.5 to 7.8 log_10_ CFU/g. Initially, 344 isolates were obtained and pulsed-field gel electrophoresis (PFGE) profiling allowed the reduction of the collection to 101 strains. Among them, multilocus sequence typing (MLST) profiling showed the presence of 32 different sequence types (ST). Globally, most of the STs to hospital-adapted high-risk clones and belonged to clonal complexes (CC) associated to the hospital environment, such as CC2. The virulence gene most commonly detected among the strains was *alt*E. High resistance rates to macrolides and aminoglycosides were detected and 64% of the strains harboured the *mec*A gene, which was codified in SCC*mec* types. Our results indicates the existence of a complex and genetically diverse *S*. *epidermidis* population in the NICU environment. A better knowledge of *S*. *epidermidis* strains may help to devise strategies to avoid their conversion from symbiont to pathobiont microorganisms in the NICUs.

## Introduction

*Staphylococcus epidermidis* is probably the most ubiquitous microorganism in human skin and mucosal surfaces [1,2]. This species seems to play a beneficial role in infants by inhibiting virulent pathogens and educating the innate immune system [3]. Several studies have shown that *S*. *epidermidis* is the predominant bacteria in colostrum and milk from healthy women [4–10] and there is a mother-to-infant transmission through breastfeeding [11]; in fact, its presence is known to be a differential trait of the fecal microbiota of breast-fed infants when compared to that of formula-fed ones [10, 12–16], being already present in the first meconium obtained from both term and preterm breastfed neonates [17, 18]; however, coagulase-negative staphylococci (CNS) have received a marginal attention regarding their role in the early colonization of the infant gut in contrast to lactobacilli, bifidobacteria and other gut-associated strict anaerobes.

The commensal or beneficial role of *S*. *epidermidis* in healthy hosts, including infants, may dramatically change in preterm neonates. Several factors in the neonatal intensive care unit (NICU), such as the widespread use of medical devices (catheters, enteral feeding tubes, mechanical ventilation…) where they can rapidly form thick biofilms, the selective pressure due to antibiotics or the immunocompromised status of the host, can explain the conversion of *S*. *epidermidis* from a symbiont member of the human microbiota to a very relevant opportunistic infectious agent [3]. Under such circumstances, this species has emerged as the predominant agent causing neonatal late-onset sepsis (LOS) in very low birth weight (VLBW) preterm neonates both in developed and developing countries [19–23]. Generally, LOS by CNS is associated to lower mortality rates when compared to other LOS agents [24]. However, beyond sepsis, *S*. *epidermidis* is associated with several neonatal morbidities, such as bronchopulmonary dysplasia, white matter injury, necrotizing enterocolitis and retinopathy of prematurity, which exert short- and long-term detrimental effects on neonatal outcomes [25, 26].

In a previous work, we studied the microbiota of feces from preterm neonates by using both culture-dependent and -independent techniques [18]. The dominant species found were *S*. *epidermidis*, *Staphylococcus aureus*, *Enterococcus faecalis*, *Enterococcus faecium*, *Serratia marcescens*, *Klebsiella pneumoniae* and *Escherichia coli*. Subsequently, most of the dominant species were characterized and a high proportion of antibiotic-resistant high-risk clones was detected during the NICU admittance [27]. However, although *S*. *epidermidis* was the main species taken in account to the percentage of carrying infants, isolates from this species were initially excluded from the first analysis due to the high number of isolates and because they seemed to have a particularly wide genetic diversity. In this context, the objective of this study was to characterize a collection of *S*. *epidermidis* strains isolated from meconium and feces of preterm infants.

## Material and methods

### Patients and sampling

Twenty-six preterm infants (gestational age <32 weeks and/or weight <1,500 g) born between October 2009 and June 2010 in the University Hospital Doce de Octubre (Madrid, Spain) were included in the study. Preterm infants with malformations, metabolic diseases or severe conditions were excluded. The local ethic committee (CEIm Hospital Universitario 12 de Octubre) approved the study (reference 09/157), and parents signed informed consent. All infants were fed with human milk (pasteurized donor milk and/or their mother’s own milk [MOM]) and, occasionally, with preterm formula. The fortifier FM 85 (Nestlé, Vevey, Switzerland) was used from approximately the fourth week of life. The high heterogeneity in feeding patterns prevented the formation of well-defined feeding groups.

First spontaneously evacuated meconium and fecal samples were collected weekly during the first month of life. Feeding samples were collected after their pass through the enteral feeding tubes from either the syringe or the extension set connected with the orogastric or nasogastric feeding tube. Extension sets were routinely replaced and discarded every 24 h, thus during this period different feed types could pass through the same tube. In contrast, the nasogastric feeding tubes were remained inserted for up to 4 days. Contamination of feeding tubes and the connector with the extension set was reviewed and confirmed in a previous work [28]. Preparation of the feedings previous to their pass through the enteral feeding system was as follows: (a) MOM was extracted using an electric pump and stored either refrigerated (5°C) for maximum of 24 h, or frozen (-20°C) up to 3 months; (b) donor milk was pasteurized (62.5°C, 30 min) and stored frozen (-20°C) after collection up to 3 months; and (c) commercial sterilized formula milk was ready-to-use in individual doses. All feedings were incubated for 10 to 15 min at 37°C to 40°C before their administration. After collection, the samples were immediately stored at −80°C until analysis.

### Culture-based analysis, bacterial identification and genotyping

Adequate dilutions of meconium, feces and feeding samples obtained during the NICU stay were spread onto Columbia Nalidixic Acid (CNA, BioMérieux) agar plates. Plates were aerobically incubated at 37°C for up to 48 h. After bacterial counting, at least one representative of each colony morphology were analyzed by optical microscopy and those isolates compatible with staphylococcal morphology were identified by species-specific PCR based on the *dnaJ* genes with primers J-StGen (5’-TGGCCAAAAGAGACTATTATGA-3’), J-StEpi (5’-CCACCAAAGCCTTGACTT-3’) [10]. Bacterial identification was also confirmed by matrix-assisted laser desorption/ionization time-of-flight (MALDI-TOF) mass spectrometry in a Vitek-MS instrument (BioMérieux).

In order to avoid repeated isolates, DNA from the *S*. *epidermidis* isolated colonies was extracted following the protocol of Ruiz-Barba et al. [29] and submitted to randomly amplified polymorphic DNA PCR (RAPD-PCR) using the primer OPL5 (5’-ACGCAGGCAC-3’) as described by Rodas et al. [30]. At least a representative isolate from each RAPD profile per sample was selected to perform PFGE in a CHEF DR II apparatus (Bio-Rad, Birmingham, UK). *Sma*I-digested fragments were separated with the electrophoresis conditions of 5 to 15 s for 10 h and 15 to 60 s for 13 h. A dendrogram analysis of PFGE profiles was performed using the UPGMA method based on Dice similarity index and the INFO QUEST software (BioLine). Finally, the *S*. *epidermidis* MLST scheme was applied for PFGE-unrelated strains of *S*. *epidermidis* (http://www.mlst.net). Genetic diversity was represented by the Minimum Spanning Tree algorithm using the Phyloviz software (http://www.phyloviz.net).

### Screening for potential virulence and antibiotic resistant determinants

Assessment of genes encoding virulence factors was carried out with the same set of strains that were submitted to MLST analysis. The tested genes included *ica*D, *fbe* and *alt*E (which encode proteins involved in adhesion and biofilm production) [31] and *mec*A (resistance to methicillin) [32]. The staphylococcal cassette chromosome *mec* (SCC*mec*) was determined by using a typing procedure involving a PCR amplification (*ccr*B genes) followed by restriction fragment length polymorphism (RFLP) analysis using endonucleases *Hinf*I and *Bsm*I [32]. SCC*mec* elements are classified into types by a hierarchical system, considering types I, II and III have a hospital-acquired origin, whereas type IV is associated to the community.

### Antimicrobial susceptibility testing

All the *S*. *epidermidis* strains submitted to MLST analysis were also tested for antibiotic susceptibility testing. Susceptibility to penicillin, oxacillin, gentamicin, tobramycin, levofloxacin, erythromycin, clindamycin, linezolid, daptomycin, teicoplanin, vancomycin, phosphomycin, fusidic acid, mupirocin, rifampicin and cotrimoxazol was assessed in a MicroScan WalkAway (Beckman Coulter) compact equipment. The breakpoints were selected according to the guidelines of the Clinical and Laboratory Standards Institute [33].

### Statistical analysis

The statistical analysis was performed using R 2.15.3 (R-project, http://www.r-project.org). Data not normally distributed were represented as medians and interquartile ranges (Q1 and Q3); on the other hand, normally distributed data were expressed as means and 95% confidence interval (95% CI). The Kruskal-Wallis test was used to evaluate the differences between sampling times in non-normal data. Fisher’s exact test was used to compare proportions. In all cases, *P* values of <0.05 were considered to be significant.

## Results

### Characteristics of premature infants

Demographic and clinical data of the infants are provided in Table 1. Mean gestational age and birth weight were 27.7 weeks and 1,167 g, respectively. Most of them (92%) received antibiotic treatment during their hospital stay. Some of them (42%) received a combination of ampicillin and gentamicin as a prophylactic antibiotherapy while these and other antimicrobials (amphotericin B, amikacin, cloxacillin, erythromycin, fluconazole, fluorocytosine, meropenem, micafungin, teicoplanin, vancomycin) were occasionally used against a confirmed or suspected infection. Approximately half of the infants (54%) were born by Cesarean section and, consequently, their mothers received prophylactic or presurgical antibiotics per established clinical protocol. Respiratory disorders were common among our population since 21 of the newborns required continuous positive airway pressure (CPAP), 20 of them needed oxygen therapy and 16 of them required the use of mechanical ventilation, with median durations of 9, 32 and 8.5 days, respectively. Nearly all infants required parenteral nutrition at birth for a median duration of 7.5 days while all of them were fed with enteral feeding tubes for a mean period of 59 days. Median values for NICU and hospital stays reached 42 and 64 days, respectively. 27% of the infants suffered at least one sepsis episode during their NICU admittance. *S*. *epidermidis* was identified in the 71% of the sepsis episodes and affected to 19% of the infants, either alone or together with other microorganisms.

**Table 1:** Demographic data and clinical characteristics of the preterm infants participating this study.

Characteristics			
**Infants**			26
**Gestational age (wk)**^a^			27.7 (26.6;28.7)
**Gender**^b^			
	Male		13 (50%)
	Female		13 (50%)
**Birth weight (g)**^a^			1,167 (987;1,347)
**Delivery mode**^b^			
	Vaginal		12 (46%)
	Cesarean section		14 (54%)
**Antibiotherapy (days)**^b^			
	No		2 (8%)
	Yes		24 (92%)
		<3 days	11 (42%)
		>3 days	13 (50%)
**Bronchopulmonary dysplasia**^b^			
	No		15 (58%)
	Yes		11 (42%)
**Chorioamnionitis**^b^			
	No		24 (92%)
	Yes		2 (8%)
**Sepsis**^b^	Total		7 (27%)
	Caused by *S*. *epidermidis*		5 (19%)
**Parenteral nutrition, n = 22 (days)**^c^			7.5 (5–12.8)^a^
**Enteral feeding tube (days)**^a^			59 (46;72)
**Mechanical ventilation, n = 16 (days)**^c^			8.5 (1–35.5)^a^
**CPAP, n = 21 (days)**^c^			9 (5–45)^a^
**Oxigenotherapy, n = 20 (days)**^c^			32 (2–84)^a^
**NICUs (days)**^c^			42 (18.5–77.8)^a^
**Hospital stay (days)**^c^			64 (41–90)^a^

Values expressed as:

^a^Mean (95% CI);

^b^n (%);

^c^Median (IQR).

### *S*. *epidermidis* from feces and feeding systems

The microbial culture of fecal samples yielded *S*. *epidermidis* colonies in all infants, although not in all samples. *S*. *epidermidis* was frequently detected in the meconium samples (38%), although its presence notably increased in feces collected at day 7 after birth (89%). Subsequently, its fecal presence decreased progressively until day 28, when only 27% of the infants harbored it (Table 2).

**Table 2:** Isolation and quantification (log_10_ cfu/g) of *S*. *epidermidis* in meconium and fecal samples the recruited infants.

			Meconium (n = 16)	1st week feces (n = 18)	2nd week feces (n = 23)	3rd week feces (n = 24)	4th week feces (n = 11)	P value
Total		n (%)	6 (38%)	16 (89%)	13 (57%)	12 (50%)	3 (27%)	
		Microbial Counts Median (IQR)	6.80 (4.80–8.80)	7.85 (7.00–8.38)	6.70 (6.30–7.30)	6.50 (5.93–7.53)	7.70 (7.65–8.50)	0.098^a^
Antibiotics previous to sample collection	YES	n (%)	3 (50%)	15 (94%)	1 (8%)	3 (25%)	0 (0%)	
		Microbial Counts Median (IQR)	6.60 (4.65–8.10)	7.70 (7.02–8.45)	5.00 (5.00–5.00)	7.65 (7.18–7.91)	-	0.000^a^
	NO	n (%)	3 (50%)	1 (6%)	12 (92%)	9 (75%)	3 (100%)	
		Microbial Counts Median (IQR)	7.93 (5.66–8.70)	8.00 (8.00–8.00)	7.13 (6.62–7.37)	6.39 (5.62–7.53)	7.54 (6.09–9.19)	0.225^a^

^a^Friedman test.

In the positive samples, the *S*. *epidermidis* counts varied between 6.5 and 7.8 log_10_ CFU/g over the studied period. In order to evaluate the effect of antibiotherapy specific to Gram-positive bacteria on the *S*. *epidermidis* counts over time, samples were subdivided in those that were collected after an antibiotic treatment or not. In this case, *S*. *epidermidis* counts varied from 5.0 to 7.7 log_10_ CFU/g (p.value 0.000) and 6.4 and 8.0 in samples collected when antibiotics were administered in the previous days or not respectively (Table 2). An increase in the concentration values from meconium to day 7 feces was observed; then, the concentrations decreased slowly and rose again at the end of the first month of life of the infants. When fecal bacterial counts were related to demographic and clinical data, *S*. *epidermidis* colonization tended to be higher in those infants with longer NICU stay, requiring more days of CPAP or mechanical ventilation, and born by Cesarean section, although the values did not reach a statistically-significant level. Interestingly, the *S*. *epidermidis* bacterial counts were slightly lower in those infants suffering sepsis caused by *S*. *epidermidis*, however is important to remark that these infants were treated with antibiotics (combinations of Teicoplanin with Vancomycin, Eritromycin, Gentamicin, Meropenem and/or Amikacin) at the moment of sample collection (Table 3).

**Table 3:** Relationships between *S*. *epidermidis* colonization (counts expressed as log_10_ CFU/g) and the main clinical and demographic data.

Parameter		Positive samples and counts		P value
		n (%)	Microbial Counts Median (IQR)	
Gestational age	< 28 weeks	16 (62%)	7.91 (6.47–8.35)	0.958
	> 28 weeks	10 (38%)	7.51 (6.61–8.56)	
Birth weight	< 1000 g	11 (42%)	7.98 (7.03–8.37)	0.391
	> 1000 g	15 (58%)	7.46 (6.18–8.48)	
Delivery mode	Vaginal	12 (46%)	7.47 (6.67–7.94)	0.291
	Cesarean section	14 (54%)	8.31 (6.20–8.71)	
Antibiotherapy (days)	No Ab or <3 days	10 (38%)	7.46 (6.61–8.12)	0.399
	>3 days	16 (62%)	7.95 (6.47–8.66)	
Mechanical ventilation	YES	16 (62%)	7.95 (7.20–8.50)	0.246
	NO	10 (38%)	7.08 (5.98–8.13)	
CPAP	< 7 days	13 (50%)	7.36 (5.78–8.32)	0.238
	> 7 days	13 (50%)	7.98 (7.45–8.42)	
Parenteral nutrition	< 7 days	15 (58%)	7.57 (6.64–8.68)	0.736
	> 7 days	11 (42%)	7.92 (6.24–8.31)	
Enteral feeding tubes	< 45 days	11 (42%)	7.46 (6.70–8.48)	0.835
	> 45 days	15 (58%)	7.92 (5.74–8.37)	
CVN	< 7 days	13 (50%)	7.57 (6.70–8.64)	0.700
	> 7 days	13 (50%)	7.90 (5.78–8.32)	
NICU stay	< 45 days	15 (58%)	7.45 (6.18–8.11)	0.119
	> 45 days	11 (42%)	8.30 (7.64–8.57)	
Hospital stay	< 60 days	13 (50%)	7.46 (6.70–8.64)	0.959
	> 60 days	13 (50%)	7.92 (5.78–8.32)	
Predominance of MOM feedings	No	5 (19%)	7.90 (7.57–8.30)	0.625
	Yes	21 (81%)	7.46 (5.78–8.42)	
*S*. *epidermidis* Sepsis	No	21 (81%)	7.90 (6.70–8.64)	0.228
	Yes	5 (19%)	6.70 (5.78–7.92)	
Sampling time	Meconium (n = 16)	6 (38%)	6.80 (4.80–8.80)	0,098*
	1st week feces (n = 18)	16 (89%)	7.85 (7.00–8.38)	
	2nd week feces (n = 23)	13 (57%)	6.70 (6.30–7.30)	
	3rd week feces (n = 24)	12 (50%)	6.50 (5.93–7.53)	
	4th week feces (n = 11)	3 (27%)	7.70 (7.65–8.50)	

*Friedman test.

In relation to the feeding samples after their pass through the enteral feeding system, *S*. *epidermidis* was present in 84% of the MOM samples while the detection frequency was significantly lower in donor milk and formula samples (42% and 14%, respectively). *S*. *epidermidis* counts were also considerably higher in MOM samples (Table 4).

**Table 4:** *S*. *epidermidis* isolated from the feeding samples after their pass through the enteral feeding tubes. Counts expressed as log_10_ CFU/g.

	MOM (n = 58)	Donor milk (n = 19)	Formula (n = 7)	P value
n (%)	49 (84%)	8 (42%)	1 (14%)	0.065^a^
Microbial Counts Median (IQR)	4.77 (4.22–5.28)	3.09 (2.15–4.63)	2.00	0.025^b^

^*a*^Fisher test;

^*b*^KW test.

### Genetic diversity

Initially, 344 *S*. *epidermidis* isolates were obtained from 92 fecal and 84 feeding samples. After RAPD analysis, one representative of each RAPD profile per sample was selected for further analysis and, subsequently, 173 isolates were submitted to PFGE analysis. PFGE profiling allowed the selection of 101 strains, including one representative of each different PFGE profile per infant. This final collection was genetically characterized using the MLST scheme.

**Fig 1:**
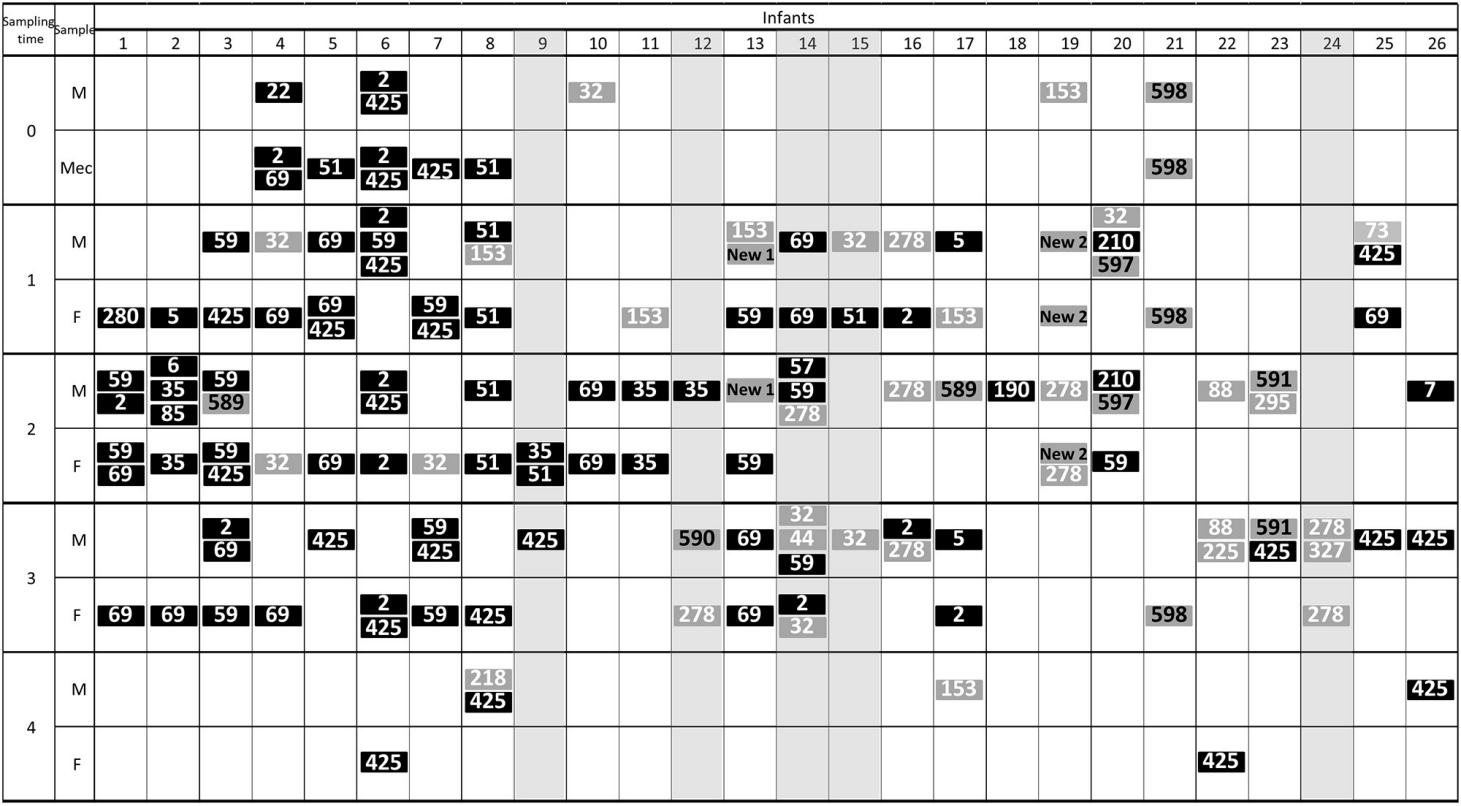
ST clones colonizing infants and the food samples used to feed them (after their pass through the enteral feeding tubes) during the first month of life. Sequence types (ST) clones associated with hospital environment are represented in black and those belonging to community in grey. Clones recently described are represented in grey with the clone number in black.

The 101 strains were distributed in 32 different sequence types (ST). The STs more frequently isolated from feces were ST69 (8 infants), ST425 (6 infants), ST59 (5 infants), and STs 2 and 51 (4 infants each), while those more widespread among the feeding samples were ST425 (9 samples), and STs 2, 32, 59 and 69 (5 samples each). Globally, the dominant STs in fecal samples were also majoritarian in the feeding ones. According to MLST profiling, the highest genetic diversity was related to MOM samples (30 different STs from 169 isolates), whereas in the fecal samples 12 different STs were identified from 103 isolates; in addition, up to 38% of the MOM samples contained 3 different ST strains while this percentage was lower (23%) in the fecal samples (Fig 1). MLST typing also revealed the transference or sharing of strains, such as ST59 in infants 1 and 3, ST35 in infants 2 and 11, ST69 in infants 5, 10, 13 and 14, ST32 in infants 4 and 14, or ST278 in infants 19 and 24, between fecal and feeding samples (Fig 1).

**Fig 2:**
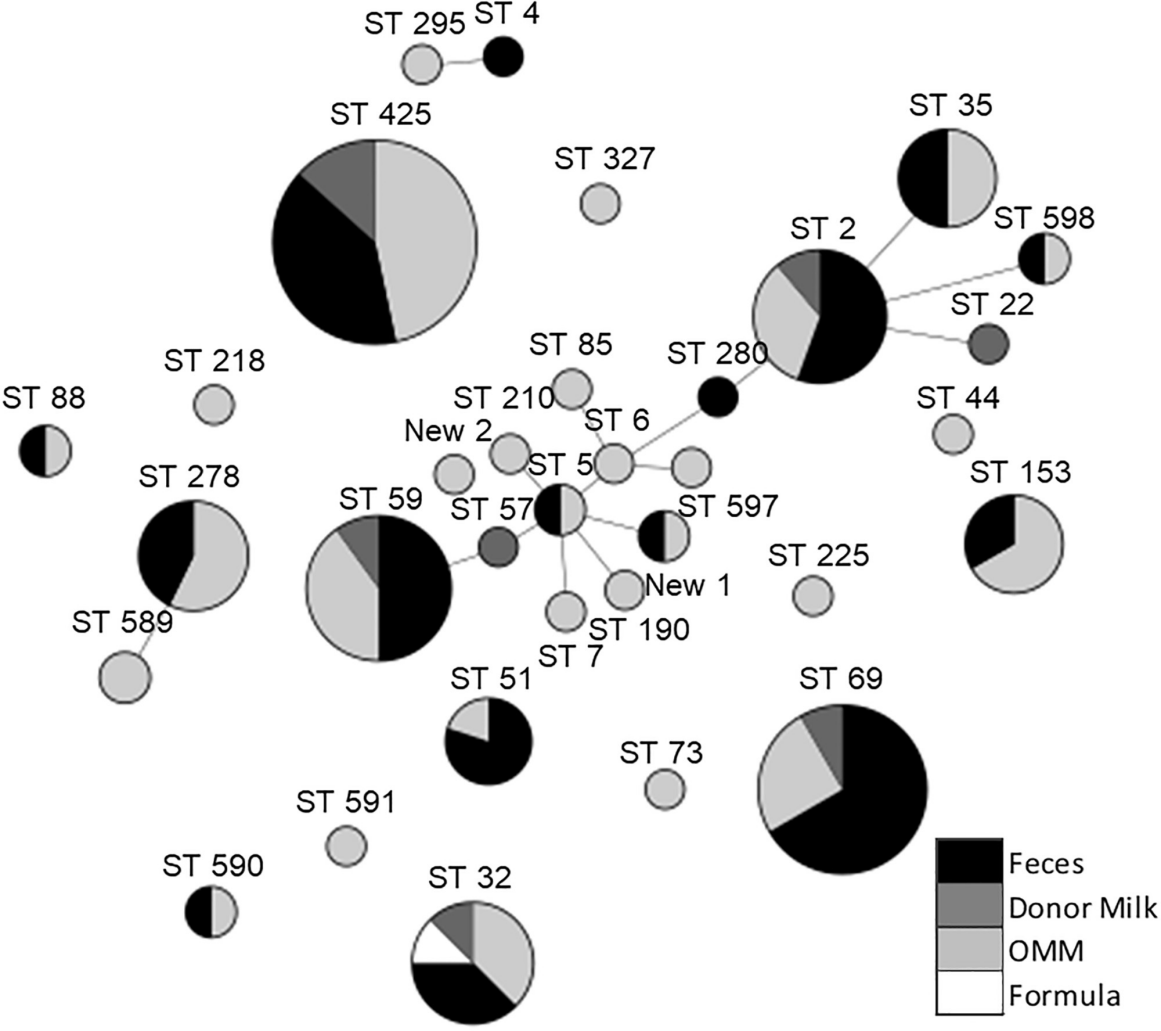
MLST genetic diversity using minimum spanning tree algorithm. Each circle represents a different MLST clone; size depends on the number of isolates in the group. Clone number is indicated above circle; Circles are colored depending on the frequency of the clone in the analyzed samples: black represent frequency in feces, light grey in MOM, dark grey in donor milk and white in formula milk samples. Clones considered to be of high risk are highlighted in bold. Genetically related sequence types (STs) are connected by grey lines.

Globally, 75% of STs detected in this study belonged to hospital-adapted high-risk clones although this percentage decreases to 53% when only milk samples are considered (Fig 1). Infants suffering a bacteremia episode caused by *S*. *epidermidis* were colonized by, at least, one strain of *S*. *epidermidis* belonged to high risk clones (Fig 1), with the exception of infant 24. From the 32 STs detected 13 belonged to clonal complexes (CC) associated to the hospital environment, as CC2, CC5 and CC6 (Fig 2). However, 7 STs had not been previously described; the new STs 589–591, 597 and 598 were assigned to five of them. The remaining two novel STs presented undescribed mutations on some of the studied alleles and their code assignment is still pending. The non-previously reported STs were mostly from MOM and only two of them (ST598 and STNew2), were also detected in fecal samples.

### Presence of potential virulence genes and antimicrobial susceptibility

**Fig 3:**
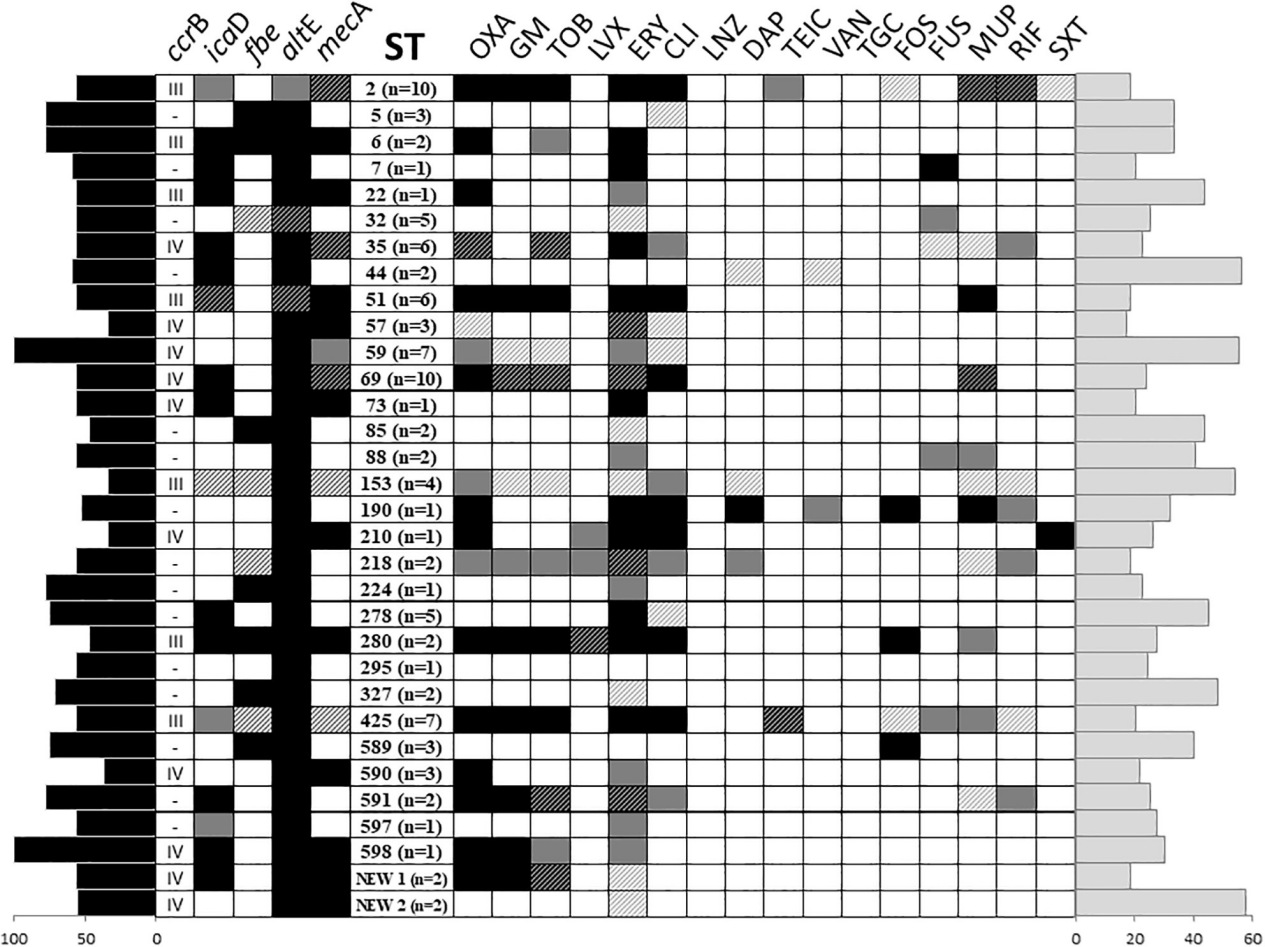
Profiles of antibiotic susceptibility and virulence factors in the different ST clones. At least one different sequence types (ST) per infant is represented in each line. Results are represented in function of frequency. Black squares means 100% of the isolates were resistant to an antibiotic or present a virulence determinant, while black and grey striped boxes means 75–99%, grey squares 50–74%, grey and white striped squares 25–49% and white boxes 0–24%. Black bars in the left side of the figure represent the percentage of virulence genes present in each ST while the grey bars on the right side of the figure shows the percentage of antibiotic resistances per ST. OXA: Oxacillin; GM: Gentamicin; TOB: Tobramycin; LVX: Levofloxacin; ERY: Erythromycin; CLI: Clindamycin; LNZ: Linezolid; DAP: Daptomycin; TEIC: Teicoplanin; VAN: Vancomycin; TGC: Tigecycline; FOS: Fosfomycin; FUS: Fusidic Acid; MUP: Mupirocin; RIF: Rifampicin; SXT: Trimethoprim-sulphamethoxazole.

The results of the screening for potential virulence genes and antimicrobial susceptibility are presented in Fig 3. The genes most commonly detected in the isolated strains were *alt*E (98%), involved in adhesion and biofilm production, and *mec*A (55%), implicated in methicillin resistance. Type III (28.4%) and IV (71.6%) SCC*mec* were identified in 16 and 29 isolates from fecal samples and 14 and 19 isolates from the enteral feeding system respectively.

Regarding antibiotic resistance, most of the *S*. *epidermidis* strains exhibited resistance to macrolides (70% to erythromycin and 56% to clindamycin), tetracycline (72%), methicillin (64%), aminoglycosides (62% for amikacin and tobramycin and 54% for gentamicin), and teicoplanin (22%) whereas almost all remained susceptible to vancomycin, tigecycline, daptomycin, quinolones and linezolid. A single isolate grouped on the ST2 lineage was resistant to all tested antibiotic including linezolid, daptomycin, fosfomycin, and cotrimoxazol. Globally, the strains with the highest virulence potential were those belonging to the STs 280, 2, 425, 190, 51 and 69, which were also resistant to oxacillin and clindamycin, and showed different resistance levels to gentamycin, tobramycin and mupirocin (Fig 3).

## Discussion

In this study we use phenotyping and genotyping techniques to determine the diversity and virulence of *S*. *epidermidis* isolates obtained from fecal samples of preterm infants during their hospital stay and from the different types of feeds (MOM, donor milk and adapted formula) collected after their pass through the enteral feeding tubes.

*S*. *epidermidis* was relatively frequent in meconium samples (~40%), but the detection frequency notably increased (~90%) in the fecal samples collected 7 days after birth. A pioneering study using a culture-dependent approach revealed that *S*. *epidermidis* was often present in aseptically-collected meconium from term neonates and suggested that gut colonization may start *in utero* during late pregnancy under physiological conditions [10]. Although the issue is still controversial and the subject of an intense scientific debate [34, 35], the results of subsequent studies that applied culture-dependent techniques to study meconium microbiota were similar, both in term [36–39] and preterm neonates [18]. The decrease in the fecal CNS populations after the first weeks of life has been previously reported [40–44].

In relation to the feeding samples, MOM contain their own microbiota while donor milk and preterm formula are usually sterile before their administration through the enteral feeding tubes. Studies carried in the last years have shown that human milk contains a site-specific microbiota and represents a continuous supply of commensal and potentially probiotic bacteria to the infant gut [5]. Most culture-dependent and–independent studies on the human milk microbiota have found that CNS, and particularly *S*. *epidermidis*, is the dominant species in this biological fluid [5, 8–10, 45–48]. Once in the infant gut, these bacteria drive the assembly of a healthy infant gut microbiota [49] and may play several functions, contributing to the infant metabolism, protection against infections, immunomodulation or neuromodulation [5].

The different milk types were subsequently administered through enteral feeding tubes which are well-suited devices for the growth and enrichment of high risk hospital-associated clones, which subsequently act as reservoirs of such clones [28, 50–52]. In preterm neonates, gastric pH values are closer to neutrality while the pylorus is more relaxed than in adults; this allows the entry of gut bacteria to the gastric compartment. Later, the widespread NICUs practice of aspirating and measuring the residual gastric content before administrating the next feeding leads to the contamination of the upper parts of the enteral feeding systems, including the external parts, with the high risk clones that characterize the preterm gut microbiota. Since feeding tubes are usually placed in the digestive tract of neonates for 24–72 h and are kept at a temperature optimal for the growth of many facultative anaerobic bacteria (CNS, enterobacteria…), such microorganisms have enough time to form thick mix biofilms in the internal surface of the tubes [28]. This may explain the presence of *S*. *epidermidis* in a percentage of donor milk and infant formula samples after their pass through such feeding systems; however, other sources, including healthcare workers and family members, can not be discarded and such possibility should be addressed in future studies.

The results of this study indicate that *S*. *epidermidis* population was genetically more diverse among MOM samples than among the fecal samples; this fact may reflect the coexistence of strains from both the human milk microbiota and the reservoir (enteral feeding tubes) when MOM passes through the enteral feeding system. Other contamination sources, such as endotracheal intubation or the hands of healthcare workers cannot be discarded. Later, the NICU environment may exert a role in selecting only those human milk strains which genome flexibility allow them to colonize the gastrointestinal tract of preterm neonates during their stay in the NICUs; as a result, the diversity of *S*. *epidermidis* strains is narrower in the feces of the infants. It must be highlighted that *S*. *epidermidis* is a bacterial species suited with an extraordinary genetic flexibility, and can employ a multitude of mechanisms to become adapted to the changing environment [3, 53]. A study comparing 200 *S*. *epidermidis* strains isolated from women with lactational mastitis with 105 isolates from milk of healthy women showed that the number of strains that contained the biofilm-related *ica*D gene and that showed resistance to oxacillin, erythromycin, clindamycin and mupirocin was significantly higher among the strains isolated from the mastitis milk [31]. However, the same study also revealed that potentially pathogenic strains could also be isolated from some samples from healthy women. Another study confirmed that human milk from healthy women may be a reservoir of strains containing potential virulence factor and displaying (multi)resistance against clinically-relevant antibiotics [54]. In this context, most of the clones found in this work were precisely those enriched in virulence factors and antibiotic resistances (ST2, ST51 and ST425), showing higher adaptation to the NICU environment and, therefore, an enhanced capacity for persistence and dissemination. More specifically, a high proportion of the *S*. *epidermidis* strains exhibited resistance to ß-lactams, macrolides and aminoglycosides. In addition, almost all the methicillin-resistant isolates were also biofilm producers and predominantly carried cassettes type III and IV, which is in agreement with previous reports [3, 20, 55–57]. Both antibiotic (multi)resistance and biofilm production capacity are common characteristics in strains (mainly CC2 ones) adapted to hospital environments [58, 59].

As previously stated [3], *S*. *epidermidis* strains may play quite different roles (from “*guarding angel to pathogenic devil*”) in the neonatal setting. A better knowledge of the predominant *S*. *epidermidis* clones (virulence factors, mechanism of biofilm formation in medical devices, mechanisms of immune-evasion, antibiotic resistances, specific sensitivity to phages, synergism or antagonism with other members of the preterm gut microbiota, CRISP systems…) that are particularly prevalent in NICUs is required in order to improve their control and, as a consequence, preterm health outcomes at the short and long terms. Microbiome-based strategies, including the restoration of the “safe” CNS population present in MOM when a neonate must be fed with pasteurized donor milk or formula have already been suggested [60]. CNS selected on the basis of a strain-by-strain rigorous safety assessment can be particularly useful to reduce the acquisition of undesired pathogens by infants, including preterm neonates, exposed to NICUs environments. It has been proposed that *S*. *epidermidis* and other CNS may have a probiotic function by preventing colonization of the host by more severe pathogens, such as *S*. *aureus* [2]. In fact, a future strategy to eradicate *S*. *aureus* from the mucosal surfaces has already been postulated based on their *in vitro* inhibition by *S*. *epidermidis* [61, 62] A better knowledge of NICU-associated *S*. *epidermidis* strains may help to devise strategies to avoid their conversion from symbiont to pathobiont microorganisms.

## Abbreviations

CNS: Coagulase-negative staphylococci
LOS: Late-Onset Sepsis
MOM: Mother’s Own Milk
NICU: Neonatal Intensive Care Unit
VLBW: Very Low Birth Weight
